# Integrative assessment of the effects of ventilation systems on economic efficiency, milk production, and reproductive performance in dairy cows

**DOI:** 10.3389/fvets.2025.1713828

**Published:** 2025-12-04

**Authors:** Arzu Peker, Luisa Magrin, Severino Segato, Chiara Mondin, Şükrü Orkan, Nurşen Öztürk, Flaviana Gottardo, Samuele Trestini

**Affiliations:** 1 Department of Animal Health Economics and Management, Faculty of Veterinary Medicine, Ankara University, Ankara, Türkiye; 2 Dipartimento di Medicina Animale, Produzioni e Salute, Università degli Studi di Padova, Legnaro, Italy; 3 Dipartimento di Territorio e Sistemi Agro-Forestali, Università degli Studi di Padova, Legnaro, Italy; 4 Department of Animal Breeding and Husbandry, Faculty of Veterinary Medicine, Istanbul University-Cerrahpasa, Istanbul, Türkiye

**Keywords:** heat stress, ventilation system, milk production, reproduction, economic analysis

## Abstract

Due to global warming, temperate regions are increasingly experiencing heat waves, which negatively impact dairy cow welfare, productivity, and farm profitability. Ventilation systems (VSs) are a common heat mitigation strategy, despite their high initial investment cost. This study investigated the effects of heat stress (HS) and VSs on dairy cows’ milk yield and whether VSs guarantee an economic benefit for farmers. The trial involved four dairy farms over 3 years: 2 years before and one after VS installation. We conducted an observational within-farm pre-post study, using two pre-installation years and 0.5–1 post-installation year per farm. The following outcomes were analyzed at the herd level: daily milk yield, biweekly milk quality, and monthly reproductive metrics. The temperature-humidity index (THI) was calculated daily as a measure of HS and categorized as follows: comfort (<72), mild discomfort (72–79), discomfort (80–84), and alert (>84). Economic sustainability was assessed through partial budget analysis, accounting for additional feed, labor, and electricity costs. The presence of VSs was associated with a significant increase in cows’ milk yield across all THI conditions (*p* < 0.001). Fat and protein contents decreased following VS installation, consistent with the observed increase in milk yield. However, their values were significantly lower under the most critical THI classes (*p* < 0.001). Linear somatic cell count (SCC) scores were higher under the discomfort and alert THI classes in the absence of VSs, whereas they decreased slightly across THI classes when VSs were used (*p* < 0.001). The duration of lactation, days open, and number of services per pregnancy reached their highest values under THI alert conditions in the absence of VSs and were significantly reduced following the implementation of VS (*p* < 0.001). Increased milk income with VS use was €12.39/day/cow under mild discomfort, €12.23 under discomfort, and €12.08 under alert conditions. The results showed wide variability in economic outcomes across farms and THI classes. Although differences in VS management prevented a definitive conclusion regarding return on investment (ROI), the findings suggest positive effects on cows’ productivity and farm profitability. However, a definitive ROI could not be stated due to heterogeneity in fan size/spacing, cows-per-fan coverage, and farm-specific post-installation durations. Therefore, future cost–benefit analyses should consider additional factors to fully evaluate VS investments.

## 1 Introduction

Dairy production has been greatly affected by global warming, especially in temperate countries, in recent years. One of the impacts of global warming on the dairy industry is heat stress (HS), which can negatively affect the productivity and reproductive health of dairy cows (1), leading to reduced milk yield, higher mortality rates, poor welfare conditions, and decreased productivity and reproductive performance (2). Although each of these deteriorations is significant, reduced milk production caused by heat stress can result in substantial economic losses, particularly in temperate regions, as milk is a major source of income for farmers (3). Another crucial factor impacted by cooling systems and environmental conditions is the reproductive performance of dairy cows. In addition to milk yield, heat stress has been shown to negatively influence reproductive parameters such as age at first calving, days open, and lactation length, leading to impaired herd productivity (4, 5). Moreover, heat stress has been associated with an increased somatic cell count (SCC), indicating a higher risk of mastitis and reduced milk quality (6). These negative outcomes not only compromise animal welfare but also result in substantial economic losses for farmers (7, 8).

Due to increasing concerns about heat tolerance levels in dairy herds in temperate regions, there is a growing need for effective strategies to mitigate heat stress (9). These strategies involve providing shade during grazing, installing evaporative cooling systems in confined environments, and using artificial intelligence for real-time monitoring of animal welfare on farms (10, 11). Effective ventilation strategies are required to ensure regular good air quality and temperature control in dairy barns (12). Studies have indicated that applied technologies such as tunnel ventilation and evaporative cooling systems in farms may help reduce heat stress in dairy cows, improving milk production, metabolic efficiency during lactation, and overall farm profitability (10, 13). Conversely, effective cooling systems can support reproductive traits in dairy cows in hot and humid climates by maintaining continuous lactation and overall herd productivity (13). Many studies have investigated the effects of heat stress on production and reproductive parameters in dairy cows (4, 5, 14–17), but research on the impact of ventilation systems (VSs) on econometric parameters is limited (7, 8, 18, 19). Therefore, the aim of this study was to evaluate the effectiveness of ceiling fans as a heat stress mitigation strategy in improving milk yield, milk quality, and reproductive performance in dairy cows, as well as to assess whether this technique provides an economic benefit for farmers.

## 2 Materials and methods

### 2.1 Study design and data structure

We conducted an observational within-farm pre-post study across four commercial dairy farms. For each farm, the dataset included two pre-installation years and a post-installation monitoring window of 0.5–1.0 year, depending on farm-specific conditions. Analyses were performed at the herd level, including daily milk yield, biweekly milk quality (factory testing), and monthly reproductive metrics (herd-management records). The design maximizes within-farm comparability; however, the non-randomized, unbalanced post-installation windows introduce potential secular (calendar-time) trends, which we addressed by modeling temperature-humidity index (THI)-based exposure.

To test the effect of ceiling fans on the productive and reproductive performance of dairy cows, herds were continuously monitored from 2 years before (Absence) the year of installation to 6 months or 1 year later (Presence), based on the availability of data for each farm. Specifically, in farms 1 and 2, data collection was carried out from the beginning of 2016 to the end of 2018 and ceiling fan installation occurred in June 2018. In farm 3, data collection was carried out for the years 2016, 2017, and 2018, with fan installation occurring in May 2018. Finally, farm 4 was monitored from the beginning of 2015 to the end of 2018, with ceiling fans installed in May 2017. We prioritized a THI-based specification to avoid collinearity with calendar indicators (Month/Year) in this small, unbalanced panel. Farm 4 was analyzed over its full post-installation window to preserve information and precision, with inference centered on THI-based exposure contrasts rather than calendar indicators. A schematic representation of the monitoring periods for the sampled farms is illustrated in Supplementary Figure 1.

### 2.2 Dairy herds’ location, housing, and management

Dairy farms participating in the *Stalla 4.0* project, within which this study was conducted, were included in the analysis. These farms agreed to install ceiling fans for evaluation and provided complete data for the pre- and post-installation periods. The study was carried out in four dairy farms located in north-eastern Italy, having a herd size ranging from 70 to 200 dairy cows. A total of three of the four farms reared cows belonging to the Holstein breed, while one farm reared cows belonging to the Italian Brown breed. In all farms, lactating cows were kept in free-stall systems with cubicles and bedding materials such as rubber mattresses, straw, or sawdust. Replacement and dry cows were housed in deep-litter pens. Cows were milked twice daily in a milking parlor and fed *ad libitum* with a total mixed ration (TMR) prepared and delivered once a day. Based on herd size, three to five automated ceiling fans that created aerodynamic systems 3 to 5 m in diameter were installed in the sampled farms as a heat stress mitigation strategy.

Detailed information on farm location, cow housing and management, and ventilation systems (VSs) for the sampled farms is reported in Table 1.

**Table 1:** Characteristics of farm location, herd housing and management, and ventilation systems.

Parameter	Farm 1	Farm 2	Farm 3	Farm 4
Location *	Thiene (province of Vicenza)	Marano Vicentino (province of Vicenza)	San Polo di Piave (province of Treviso)	Breganze (province of Vicenza)
Breed raised	Holstein	Italian Brown	Holstein	Holstein
Herd size (cows, n)	110	130	135	200
Lactating cows (n)	50	70	70	92
Housing for lactating cows	Freestall with cubicles	Freestall with cubicles	Freestall with cubicles	Freestall with cubicles
Bedding material on cubicles	Mattresses, calcium carbonate, straw	Concrete, compacted manure, straw	Concrete, straw	Concrete, sawdust
Movement and feeding alleys	Solid concrete	Fully-slatted	Fully-slatted	Solid concrete
Cleaning system	Scraper	–	–	Scraper
Housing for dry and replacement cows	Deep litter	Deep litter	Deep litter	Deep litter
Milking system	Milking parlor	Milking parlor	Milking parlor	Milking parlor
Milking parlor type	Tandem	Tandem	Fishbone	Parallel
Feeding plan	TMR distributed once daily	TMR distributed once daily	TMR distributed once daily	TMR distributed once daily
Ventilation system installed	Four ceiling fans (3 m in diameter)	Three ceiling fans (5 m in diameter)	Four ceiling fans (3.5 m in diameter)	Five ceiling fans (5 m in diameter)
Year of installation	2018	2018	2018	2017

TMR, Total mixed ration. *Map of experimental farms: https://www.google.com/maps/d/u/0/edit?mid=1cC5IG4p6KrR5b85KvcEx8IDO9p5w7rk&usp=sharing.

### 2.3 Climate conditions

Daily environmental air temperature (T, °C) and relative humidity (RH, %) for the years 2015 to 2018 were obtained from the Official Regional Weather Agency (Agenzia Regionale per la Prevenzione e Protezione Ambientale del Veneto, Padova, Italy) from meteorological stations located within a maximum distance of 10 km from the selected farms. These climatic data were used to generate the daily maximum THI according to the formula of the National Research Council (20) as follows: (1.8 × T + 32) − (0.55–0.0055 × RH) × (1.8 × T - 26). To estimate the risk of heat stress in cows, daily maximum THI values were categorized into four classes: comfort (THI < 72), mild discomfort (72 ≤ THI ≤ 79), discomfort (80 ≤ THI ≤ 84), and alert (THI > 84), following widely used dairy thresholds to facilitate comparability. In Holstein studies, THI ≥ 72 is often treated as the practical onset of measurable production and physiological impacts (21). Earlier responses closer to THI ≈ 68–72 have also been reported in high-yield cows or arid contexts (22). Trends in average maximum temperatures and relative humidities from 2015 to 2018 are graphically presented in Supplementary Figure 2.

### 2.4 Milk yield and quality

Milk yield was recorded daily by personnel belonging to the cheese factory who were in charge of milk withdrawal throughout the entire monitoring period of the sampled farms. The total daily milk volume delivered was divided by the number of lactating cows on each farm. Milk quality (fat, protein, somatic cell contents) was analyzed and provided by the personnel of the same cheese factory every 15 days for each farm.

### 2.5 Reproductive parameters

Cows’ reproductive parameters, such as mature cow-equivalent milk production, age at the control day, age at first calving, number of lactations, lactation duration, number of services per pregnancy, days open, and dry period duration, were collected at the herd level on a monthly basis for each farm by the recording system of the Italian National Breeders Association throughout the entire monitoring periods of the sampled farms. These traits were computed as herd-month aggregates for each farm.

### 2.6 Statistical analysis

The herd was the experimental unit for all parameters, including milk yield and quality, as well as reproductive traits. For the analysis, average mature cow-equivalent milk yield and average herd age at the control day were each classified into three classes, calculating mean ± standard deviations within each farm. Milk somatic cell content was subjected to a logarithmic transformation according to Shook (23) as follows: log2 (SCC/100) + 3. Daily milk yield data were directly matched with the corresponding daily THI values recorded on the same day. Reproductive performance traits were computed as herd-level monthly aggregates, representing the overall reproductive outcomes of all lactating cows present in the herd during each month. To align these monthly data with thermal–humidity conditions, each reproductive indicator was associated with the predominant THI class recorded during the interval between each monthly check and the preceding one. Similarly, milk quality parameters, collected every 15 days, were linked to the predominant THI class of the preceding 15-day period. Milk yield, quality traits, and reproductive parameters were then analyzed using a mixed model (Proc Mixed of SAS 9.3; SAS Institute Inc.), which included THI condition (comfort; mild discomfort; discomfort; alert), ventilation system (absence or presence of VS), their interaction, mature cow-equivalent milk yield and herd age class, breed, month, and year as fixed effects, with farm as a random effect and Bonferroni adjustment. For farms 1, 2, and 3, where the period after fan installation consisted of 6–7 months, the same months before fan installation were included in the analysis, allowing for a direct comparison of the effect of ceiling fans (e.g., June, July, August, September, October, and November before fan installation versus June, July, August, September, October, and November after fan installation). For farm 4, where an entire year after ceiling fan installation was monitored, all months of the year were included in the analysis. The minimum threshold of statistical significance was set at a *p*-value of <0.05.

### 2.7 Economic analysis of ventilation systems

#### 2.7.1 Partial budget analysis

Partial budgeting was used to analyze the economic effect of adopting ceiling fans on the four dairy farms, since the investment in the cooling system was relatively small and the trial period was short. This analysis provides a consistent method for estimating the expected change in net incomes resulting from a proposed change in the farm business. In our case, the installation time of ceiling fans was different for each farm. Within the framework of partial budget analysis, the economic benefit of the changes implemented was calculated based on data from the periods before and after ceiling fans were installed on the farms for each THI class The results represent the average values across all farms for each THI class, calculated by aggregating the individual farm-level differences observed before and after the implementation of ventilation systems.

A partial budget consists of the following items, as indicated in Table 2 increased income and decreased costs on the left-hand side and increased costs and decreased income on the right-hand side, all related to the modifications associated with ceiling fans on the farms (24). As shown in Table 2, the economic benefit of the changes implemented can be calculated by identifying these four components. A negative result indicates that proceeding with the option would not be a good decision for overall farm performance, while a positive economic return indicates that the decision was advantageous. In addition to this calculation, benefits minus costs (as illustrated in Table 2), a benefit–cost ratio (B/C) was also determined. This ratio represents the amount of return on investment (ROI) for each euro spent. The quality of a partial budget analysis depends on the assumptions made during budget creation, as it relies on forecasts. To evaluate the effect of some of the expected values on profitability, sensitivity analyses were performed to identify potential false assumptions, as they help ensure accurate predictions (25).

**Table 2:** Partial budget approach used in this study.

Partial budget analysis approach			
Benefits associated with changes		Costs associated with changes	
	Increased income		Decreased income
	Decreased costs		Increased costs
+		+	
	= Total benefit		= Total cost
Total benefit−total cost = economic benefit of the changes implemented			
Expected partial budget effects in dairy farms			
Total benefit Increased income: Increased milk production due to the existence of ceiling fans Decreased costs: The costs directly related to cow comfort—those related to reproduction and health—may decrease as a result of less heat stress. Nevertheless, due to the challenges in accurately quantifying these factors, they have been excluded from the present analysis.		Total cost Decreased income: no reductions in milk production and therefore in income associated with cooling cows. Increased costs: increased feed costs as a result of the increased feed intake as well as the cooling system's operational costs and labor cost.	

Table 3 shows the economic parameters used in partial budget analysis. Changes in income and costs for each farm were analyzed by considering the relative humidity index and climatic conditions. We examined economic parameters under three different climatic conditions (mild discomfort, discomfort, and alert). Mild discomfort (72 ≤ THI ≤ 79) represents the threshold beyond which heat stress starts occurring in animals (26); therefore, in this study, we focused on the economic impacts observed when the THI exceeded 72. Ceiling fans are typically programmed to operate when the THI surpasses 72; hence, the alignment of the analysis to actual farm management practices geared toward mitigating heat stress. During data selection, we excluded data with a THI less than 72 from the economic analysis to make sure that the results only described specific aspects of the economic impact and management interventions under heat stress conditions.

**Table 3:** Parameters used in the partial budget analysis of this study.

Economic parameters	Farms overall
Milk price €/kg	0.401
Feed price, €/kg	0.20
Labor cost, €/kg	0.037
Electricity charge, €/kW	0.18
kW/fan-hr	0.78
Hours fans run/period	18 h/day
Number of lactating animals (average)	70.5
Number of fans (average)	4.5
Number of cows served by a fan	15.6
Live body weight of animals (average kg)	650
Feed (DM) intake (%)	62
Milk fat (%)	3.95

DM: Dry Matter basis: Used only to convert as-fed prices/quantities to €/kg for feed cost. Number of cows served by a fan: Number of cows within the fan’s coverage segment ÷ number of fans covering that segment; 15.6 is the across-farm, operating-day-weighted average based on as-built layouts. Feed cost: Farm surveys (2016–2018). Electricity price: CMP Impianti (0.18 €/kW) and sector references, validated against farm electricity bills (2015–2018). Milk price: CLAL/ISTAT annual averages, cheese milk, 2015–2018.

The steps undertaken in the partial budget analysis were as follows.

##### 2.7.1.1 Determining the current situation

The income and cost for each farm before ventilation systems were determined. In this analysis, income from milk sales and costs such as feed, energy, and labor were considered. The climatic conditions were classified into three groups: mild discomfort (72 ≤ THI ≤ 79), discomfort (80 ≤ THI ≤ 84), and alert (THI > 84).

##### 2.7.1.2 Description of investment or change

To prevent heat stress in the barn, ceiling fans were installed. The fans were all programmed to begin operation when the THI exceeded 72. The fans only operated during periods of heat stress to improve animal welfare and productivity by providing a cooler environment. The economic impact of this intervention was assessed using partial budget analysis, considering both the costs incurred—feed, labor, and electricity for fans—and the expected benefits that can turn into productivity.

##### 2.7.1.3 Calculating changes in incomes

The rate of increase in milk production and the additional income resulting from this increase were calculated. Additional income was calculated according to changes in milk production quantities. The milk price was constant for all farms (0,401 €/kg). The following formula was used to calculate the variation in milk production (∆Q) after the ventilation system was installed:

∆Q = (dr × Qr + da × Qa + de × Qe) × n°.
dr: Number of days in mild discomfort conditions (72 ≤ THI ≤ 79).
Qr: Quantity of milk produced (kg/day/cow) on mild discomfort days.
da: Number of days in discomfort conditions (80 ≤ THI ≤ 84).
Qa: Quantity of milk produced (kg/day/cow) on discomfort days.
de: Number of days in alert conditions (THI > 84).
Qe: Quantity of milk produced (kg/day/cow) on alert days.
n°: Number of cows served by a fan.

##### 2.7.1.4 Calculating changes in costs

The costs related to the operation of ventilation systems (electricity, feed, labor) were included. The objective of this analysis was to determine the short-term impacts of using fans; hence, only direct costs and benefits were considered in the analysis. In this context, operational costs such as electricity, feed, and labor were considered. Although this study did not take investment costs into consideration, these results could be used to perform long-term cost–benefit analyses and investment evaluations. Each cost factor was calculated based on the number of lactating animals in the farms.

Additional costs were calculated by considering changes in costs after the installation of ventilation systems.

To calculate the additional feed cost per cow, the following formula was used:

DMI (kg/day/cow) = BW × 0.0185 + EMP × 0.305 (24).
DMI = estimated dry matter intake (kg/day/cow).
BW = live body weight of the cow (kg).
EMP = energy-adjusted milk production (kg/day/cow), calculated as:
EMP = milk yield× (0.4 + 0.15 × actual fat percentage).
Milk yield (kg/day/cow) = total daily milk produced.
Actual fat percentage (%) = measured fat content in the milk sample.

ΔCa=Ca×ΔQ×dr

where,

Ca: cost of feed in €/kg of dry matter, ∆Q: increase in daily dry matter intake per cow (kg/day/cow), and dr: number of cow-days in the THI groups.

Dry matter intake (DMI) was predicted according to the ICAR Appendix (27). In our notation, EMP simply denotes the standard 4% fat-corrected milk (FCM) defined by the ICAR, and no alternative model was used. Milk yield and fat percentage were collected from farm records.

The additional labor cost was calculated using the following formula:


ΔClav=lav×ΔQ×dr

where,

lav: cost of labor €/kg, ∆Q: change in labor quantity, and dr: number of cow-days in the THI groups.

Labor cost was allocated per kilogram of milk; lav denotes the unit labor cost (€/kg milk) obtained from farm records, ΔQ represents the change in milk quantity (kg/cow/day) between the THI categories, and dr represents the number of cow-days.

To calculate the additional energy cost, the following formula was used:


Ce=(hr×dr+ha×da+he×de)×kW

where,

hr: hours of fan operation on mild discomfort days, dr: number of days in mild discomfort conditions, ha: hours of fan operation on discomfort days, da: number of days in discomfort conditions, he: hours of fan operation on alert days, and de: number of days in alert conditions) (0.18 €/kW; 18 h of use per day; power of 0.78 kw/h.

##### 2.7.1.5 Calculation of the net economic impact

For each farm, the total additional income was compared with the total additional costs. The net income impact was calculated by subtracting the total additional costs from the total additional income. To enable comparisons across farms of different sizes, all economic indicators were also expressed on a per-cow basis (€/cow/year). The benefit–cost ratio (B/C) was calculated by dividing the total additional income by the total costs, both in aggregate and on a per-cow basis.

##### 2.7.1.6 Sensitivity analysis

Sensitivity analysis was carried out to make the partial budget analysis more comprehensive and robust. This analysis supports the decision-making process by examining the impact of changes in specific variables on economic outcomes (28). It was analyzed how the benefit–cost ratio (B/C) would be affected if the milk price increased by 10 and 20% or decreased by 10 and 20%; the cost of €/kW increased by 10 and 20% or decreased by 10 and 20%; and the feed price increased by 10 and 20% or decreased by 10 and 20%.

##### 2.7.1.7 Breakeven analysis

The breakeven milk price was calculated by dividing the total costs of the cooling system by the additional milk produced with cooling. This represents the milk price at which the extra milk yield generated by cooling becomes profitable and fully offsets the costs of the cooling system (24).

##### 2.7.1.8 Income over feed costs (IOFC)

The income over feed costs (IOFC) was calculated by subtracting the feed cost with VS (€/day/cow) from the total milk income with VS (€/day/cow) (29).

## 3 Results

### 3.1 Effect on milk production and reproductive traits

**Figure 1:**
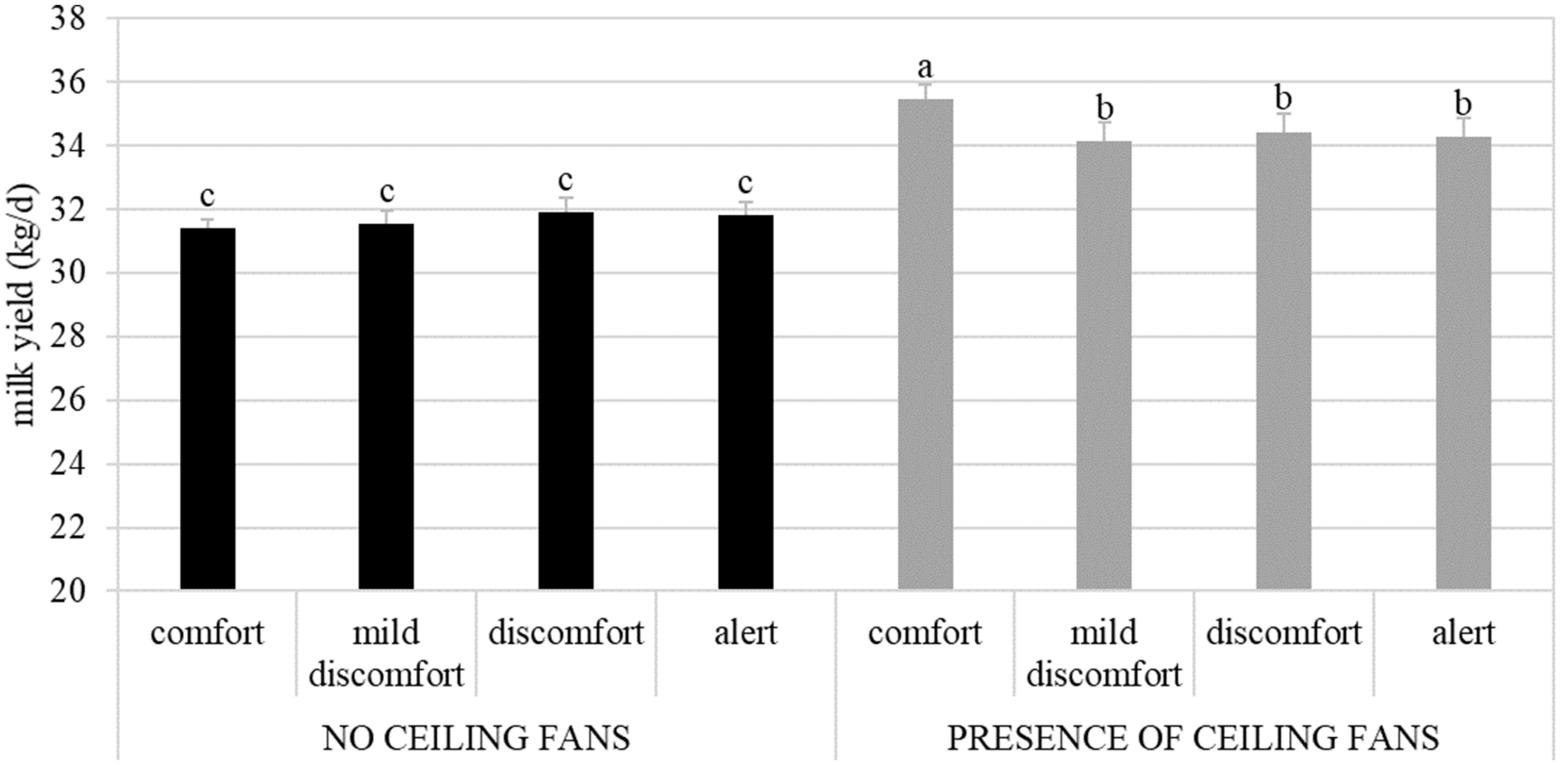
Effect of the interaction between ventilation systems (absence or presence of ceiling fans) and THI classes (comfort, mild discomfort, discomfort, and alert) on milk yield in the four dairy herds reared in Northern Italy.

The presence of VSs significantly increased milk yield in cows, especially under comfort THI conditions (Figure 1; Table 4).

**Table 4:** Effects of ventilation systems, THI conditions, and their interaction on milk yield and quality, as well as on cow reproductive parameters, in the four dairy herds reared in Northern Italy.

	Ventilation system (VS)		SEM	THI condition				SEM	*P*-value		
	Absence	Presence		Comfort	Mild discomfort	Discomfort	Alert		VS	THI	VS × THI
Milk parameters											
Milk yield (kg/d)	31.7^b^	34.6^a^	0.17	33.4^a^	32.9^b^	33.0^ab^	33.2^ab^	0.18	<0.001	0.026	<0.001
Milk fat (%)	3.91^a^	3.83^b^	0.02	3.91^a^	3.85^bc^	3.82^c^	3.87^b^	0.01	<0.001	<0.001	<0.001
Milk protein (%)	3.36^a^	3.26^b^	0.01	3.56^a^	3.31^b^	3.28^c^	3.28^c^	0.01	<0.001	<0.001	<0.001
Linear score of the SCC	3.75^a^	3.50^b^	0.03	3.73^a^	3.54^c^	3.57^bc^	3.65^ab^	0.03	<0.001	<0.001	<0.001
Reproductive traits											
Duration of lactation (days)	192^a^	167^b^	1.17	171^b^	190^a^	168^b^	198^a^	1.18	<0.001	<0.001	<0.001
Number of lactations	2.26^a^	2.24^b^	0.01	2.26^b^	2.22^c^	2.32^a^	2.21^c^	0.01	0.003	<0.001	<0.001
Age at first calving (months)	26.3^b^	27.6^a^	0.09	26.5^c^	27.5^a^	27.1^b^	26.8^b^	0.09	<0.001	<0.001	<0.001
Number of services per pregnancy	2.23^a^	2.14^b^	0.02	2.16^b^	2.16^b^	2.09^c^	2.34^a^	0.02	0.001	<0.001	0.001
Days open	146^a^	131^b^	1.00	139^ab^	140^a^	136^b^	139^a^	1.00	<0.001	0.007	<0.001
Duration of dry periods (days)	65.1^b^	66.9^a^	0.20	65.0^c^	66.1^b^	65.4^c^	67.4^a^	0.20	<0.001	<0.001	<0.001

THI: Temperature-Humidity Index. SEM: Standard Error of the Mean. Different letters indicate significant differences at a *p*-value of <0.05 within a given effect.

Milk quality traits varied according to VS adoption and THI conditions (Table 4). In particular, lower milk fat and protein contents were recorded under mild discomfort, discomfort, and alert THI conditions compared to comfort THI conditions (Table 4). The presence of the VS was associated with decreases in all these milk quality traits. The linear scores of the SCC varied significantly according to the VS × THI interaction (*p* < 0.001). Scores were higher under discomfort and alert THI classes in the absence of VSs (3.9 in comfort, 3.6 in mild comfort, 3.6 in discomfort, and 3.9 in alert), whereas they decreased slightly and remained more stable across THI classes when VSs were used (3.5, 3.5, 3.5, and 3.4, respectively). The duration of lactation shortened after VS adoption, particularly for cows under comfort conditions (before VS installation: comfort, 192 days; mild discomfort, 204; discomfort, 176; alert, 196; after VS installation: comfort, 149 days; mild discomfort, 176; discomfort, 160; alert, 181; *p* < 0.001). Age at first calving increased in the presence of VS across all THI classes compared to the absence of VS (27.6 vs. 26.3 months). The number of services per pregnancy decreased significantly after the adoption of VS and showed a peak under alert THI conditions. Days open differed significantly for the VS × THI interaction (*p* < 0.001), being shorter across all THI classes when VSs were used (before VS installation: comfort, 146 days; mild discomfort, 152; discomfort, 136; alert, 148; after VS installation: comfort, 131 days; mild discomfort, 127; discomfort, 135; alert, 129).

### 3.2 Effect on economic metrics

Table 5 presents the effects of cooling fans on milk yield, income, and costs across different THI levels. The highest milk production was recorded at THI-1 (30.89 kg/day/cow), declining to 30.50 kg/day/cow at THI-2 and 30.12 kg/day/cow at THI-3. Accordingly, milk income was 12.39€/day/cow at THI-1, 12.23€/day/cow at THI-2, and 12.08€/day/cow at THI-3.

**Table 5:** Partial budget analysis of all four dairy farms in Northern Italy.

Increased income	Cooling herd		
	THI-1	THI-2	THI-3
Milk production without VS (kg/day/cow)	29.84	29.59	29.34
Milk production with VS (kg/day/cow)	30.89	30.50	30.12
Milk income without VS (€/day/cow)	11.97	11.87	11.77
Milk income with VS (€/day/cow)	12.39	12.23	12.08
Change in increased milk yield (kg/day/cow)	0.95	0.91	0.77
Increased amount of milk production (kg/year/cow/)	40.85	35.49	18.48
Total milk production (kg/year/fan)	637.26	553.64	288.29
Total benefit (€/year/fan)	255.54	220.01	115.60
Total benefit (€/year/cow)	16.38	14.23	7.41
Costs associated with changes			
Increased costs			
Feed intake without VS (kg/DM/day/cow)	28.68	28.58	28.42
Feed intake with VS (kg/DM/day/cow)	29.14	28.82	28.72
Feed cost without VS (€/day/cow)	5.74	5.72	5.68
Feed cost with VS (€/day/cow)	5.83	5.76	5.74
Change in increased feed intake (kg/day/cow)	0.46	0.24	0.30
Additional feed costs (€/year/cow)	3.96	1.87	1.44
Total feed costs (€/year/fan)	61.78	29.17	21.69
Labor cost without VS (€/day/cow)	1.10	1.09	1.08
Labor cost with VS (€/day/cow)	1.13	1.12	1.11
Additional labor costs (€/year/cow)	0.03	0.03	0.03
Total labor costs (€/year/fan)	20.12	18.25	11.23
Energy cost (€/year/fan) **	108.67	98.56	60.65
Total costs (€/year/fan)	190.57	145.98	93.57
Total costs (€/year/cow)	12.22	9.36	5.60
Profitability of changes			
Benefits–costs (€/year/fan)	64.97	74.03	22.03
Benefits–costs (€/year/cow)	4.16	4.87	1.81
Benefit–cost (B/C) ratio (€/year/fan)	1.34	1.51	1.24
Benefit–cost (B/C) ratio (€/year/cow)	1.34	1.52	1.32
Breakeven analysis			
Breakeven milk price*	0.30	0.26	0.32
Sensitivity analysis			
B/C ratio at +10% milk price /l	1.47	1.66	1.36
B/C ratio at +20% milk price /l	1.61	1.81	1.49
B/C ratio at −10% milk price /l	1.21	1.36	1.12
B/C ratio at −20% milk price /l	1.07	1.21	0.99
B/C ratio at −10% €/kW cost	1.42	1.63	1.32
B/C ratio at −20% €/kW cost	1.50	1.77	1.41
B/C ratio at +10% €/kW cost	1.27	1.42	1.16
B/C ratio at +20% €/kW cost	1.21	1.33	1.10
B/C ratio at −10% feed price/kg	1.39	1.55	1.27
B/C ratio at −20% feed price/kg	1.45	1.58	1.31
B/C ratio at +10% feed price/kg	1.30	1.48	1.22
B/C ratio at +20% feed price/kg	1.26	1.43	1.18
Income over feed costs without VS (€/day/cow)	6.23	6.15	6.09
Income over feed costs with VS (€/day/cow)	6.56	6.47	6.34

* This is the breakeven price of milk (i.e., the price at which incremental milk production is profitable) to offset the costs of the cooling system. Installing the cooling system is therefore economically favorable, even if the dairy farm does not cover all expenditures, as long as milk prices remain at this level or higher. Calculations for each year are based on the duration of exposure to each THI class. Days are total exposure summed across farms/years. Percentages are within the THI≥72 window used for the economic analysis (comfort excluded). The total exposure amounted to 1,361 days, corresponding to 106.4 effective days after normalization: THI-1: 546 d (40.1%; eff. 42.7 d, 40.1%), THI-2: 503 d (37.0%; eff. 39.3 d, 36.9%), and THI-3: 312 d (22.9%; eff. 24.4 d, 22.9%). Totals: THI≥72 = 1,361 d; effective = 106.4 d. ** As expected, kW per effective day increases with THI due to longer duty cycles and higher fan output under warmer conditions. However, the class total reflects the scarcity of high-THI days in our panel (24.4 effective days), yielding lower aggregate energy for THI-3 despite higher per-day usage.

Additional milk yield due to cooling fans was 0.95 kg/day per cow at THI-1, 0.91 kg/day/cow at THI-2, and 0.77 kg/day/cow at THI-3, which corresponded to an annual total milk production increase of 637.26 kg per fan at THI-1, 553.64 kg at THI-2, and 288.29 kg at THI-3. The annual benefit was 255.54€, 220.01€, and 115.60€ per fan, respectively, corresponding to 16.38€, 14.23€, and 7.41€ per cow. The additional feed consumption induced by the cooling fans was 0.46 kg/day per cow at THI-1, 0.24 kg/day/cow at THI-2, and 0.30 kg/day/cow at THI-3, corresponding to annual feed costs of 61.78€, 29.17€, and 21.69€ per fan, respectively. On an overall basis, the total annual costs per fan, including labor and energy costs, were 190.57€ at THI-1, 145.98€ at THI-2, and 93.57€ at THI-3, corresponding to 12.22€, 9.36€, and 5.60€ per cow, respectively.

The calculated profitability of the cooling systems (benefit–cost) at THI-1 was 64.97€/fan (or 4.16€/cow), while it was 74.03€/fan (4.87€/cow) and 22.03€/fan (1.81€/cow) for THI-2 and THI-3, respectively. The benefit–cost ratio (€/year/fan) was 1.34 at THI-1, 1.51 at THI-2, and 1.24 at THI-3. In this case, the breakeven milk price to cover costs increased with higher THI levels: 0.30€ at THI-1, 0.26€ at THI-2, and 0.32€ at THI-3. Sensitivity analysis showed that with a 10% increase in milk prices, B/C ratios would be 1.47 for THI-1, 1.66 for THI-2, and 1.36 for THI-3. A 10% reduction in energy costs improved the B/C ratio to 1.42 at THI-1, 1.63 at THI-2, and 1.32 at THI-3. With milk price reductions of 10 and 20%, the B/C ratio decreased to 1.21/1.07 (THI-1), 1.36/1.21 (THI-2), and 1.12/0.99 (THI-3). With energy cost increases of 10 and 20%, the B/C ratio became 1.27/1.21 (THI-1), 1.42/1.33(THI-2), and 1.16/1.10 (THI-3).

## 4 Discussion

### 4.1 Effect on milk production and reproductive traits

The results show that ventilation systems can increase milk production and offset the negative impact of heat stress under diverse THI conditions. The increase in milk yield from 31.7 kg/day without VSs to 34.6 kg/day with VSs represents a significant positive effect (*p* < 0.001). This difference points to the beneficial effect of increased airflow in reducing heat stress (30) and supporting metabolic processes that are needed for milk secretion. Although this is a promising result, the 2.9 kg/cow/day increase in milk yield should be interpreted cautiously, as a within-farm pre-post design without external controls may still be affected by residual confounding from time or management changes, despite our THI-centered adjustment. A formal difference-in-difference analysis was not feasible in our study; therefore, we present operational, short-term estimates and emphasize confidence intervals over point estimates.

The THI conditions had a clear and significant impact on productivity: the highest milk yield was observed under comfort conditions (33.4 kg/day) and slightly decreased under mild discomfort (32.9 kg/day), discomfort (33.0 kg/day), and alert conditions (33.2 kg/day). This decline in milk production under heat stress might be associated with reduced feed intake, as cows experiencing stress give up productivity for thermoregulation. Das et al. (31) indicated that an increase in ambient temperature directly reduces feed intake by negatively affecting the hypothalamic center of appetite. A hot and humid atmosphere has an impact on milk quality and yield. Lower milk fat and protein contents at higher THI levels (3.91 vs. 3.87%, and 3.56 vs. 3.28% in comfort vs. alert, respectively) are indicative of poor nutrient partitioning and metabolic function. According to Kadzere et al. (32) the percentages of milk fat, solids-not-fat (SNF), and milk protein decreased by 39.7, 18.9, and 16.9%, respectively, when a lactating Holstein cow was transferred from an air temperature of 18 to 30 °C.

In our findings, reproductive performance was more variable, with ceiling fans shortening lactation duration from 192 days to 167 days (*p* < 0.001). In contrast, Khongdee et al. (33) reported that cows with cooling systems are expected to have longer lactation durations and greater milk production per lactation. These findings suggest that cows housed with VSs maintain milk production further into late lactation, allowing them to dry off earlier and thereby extending the duration of the dry period. A shorter lactation period may result from a higher likelihood of cows becoming pregnant, as indicated by the reduced number of services per conception and the shorter days-open period.

An interesting observation was that the linear score of the SCC reached its highest values across all THI classes without VSs, whereas it remained uniformly lower under all ambient conditions when VSs were adopted. Sevi and Caroprese (6) indicated that udder health issues are more common during summer temperatures because heat stress can have a detrimental effect on animal health status by changing regular physiological processes. Therefore, impaired udder health may be associated with heat stress, as a result of immune suppression caused by stress-induced hormonal alterations. These hormonal changes could affect the cow’s ability to fight infections effectively, potentially leading to increased SCC values. According to a recent study on goats, the cumulative effect of multiple challenges led to a shift in hormone release, an increase in SCC values in the milk produced, and a decrease in milk production (34). It is likely that, by mitigating heat load, VSs reduce these physiological disturbances, helping to preserve immune competence and thereby contributing to lower SCC values across all THI conditions.

### 4.2 Effect on economic metrics

Under mild discomfort, discomfort, and alert conditions, increases in daily milk production of 0.95 kg, 0.91 kg, and 0.77 kg resulted in additional incomes of 255.54€/year/fan, 220.01€/year/fan, and 115.60€/year/fan and net economic impacts of 16.38€/year/cow, 14.23€/year/cow, and 7.41€/year/cow, respectively. However, since these values are influenced by the number of days under each THI condition, they were also standardized on a daily basis to improve comparability. These results show that ventilation systems provide the highest total benefit under mild discomfort conditions while still producing a positive economic effect under both discomfort and alert conditions. Several studies have also reported that ventilation systems increase milk production and income in farms (35–37). The economic effectiveness of ventilation systems is directly linked to the additional income through improved animal comfort and productivity (38, 39). At lower THI levels, cooling systems are expected to offer the greatest advantage by alleviating heat stress in cows and supporting the preservation of milk production. Therefore, strategically optimizing ventilation systems according to climatic conditions is crucial for maximizing the net economic impact and providing sustainability in terms of profitability. However, in our study, the income increase due to improvements in reproductive parameters and SCC was not evaluated. In such a case, it could have positively affected the additional income value per fan and cow.

Ventilation systems may enhance air quality, animal welfare, and farm profitability in situations where environmental stress is minimal (40). This may be due to the fact that higher THI levels reflect extreme environmental conditions in which cooling systems, in their current form, might be insufficient to offset the extreme physiological stress experienced by cows. This is also supported by the marginal increase in milk yield per day at THI-3 (0.77 kg/d/cow) compared to THI-1 (0.95 kg/d/cow), indicating that the marginal return on investment decreases as environmental stress increases. At alert THI levels, ventilation alone is often insufficient without evaporative cooling, so incremental milk responses naturally plateau. In addition, the alert category is comparatively sparse and uneven across farms/post-windows, which increases uncertainty and can reduce estimated effects after model adjustment. Prior studies have shown that at high heat load, evaporative cooling strategies outperform ventilation alone in maintaining production and welfare (21). Therefore, for alert THI levels, we recommend using fans + soakers (or evaporative cooling) as the default strategy while reserving fans only for comfort and mild conditions. The highest levels of animal stress and adverse environmental conditions occur under alert conditions (41). The challenges dairy cows face under heat stress have been reported in studies by West (21) and Fournel et al. (42). Under these extremely adverse conditions, although ventilation systems can provide some improvement, this improvement may be more limited compared to other conditions.

Although the total benefit of the cooling system decreased progressively from THI-1 to THI-3, the highest benefit–cost ratio was recorded under THI-2 (discomfort) conditions. This reflects the point of optimal economic efficiency, where the system’s milk yield response remains substantial while operational costs are still moderate. Beyond this range (THI-3), the law of diminishing returns becomes evident and additional cooling efforts yield proportionally smaller benefits as cows reach their physiological limits under severe heat stress. Therefore, investments made under alert conditions may not provide additional benefits beyond a certain level. This low marginal benefit may result in a low B/C. Another important point is that milk production may already be very low under alert conditions. Studies by Ouellet et al. (43) and Trajchev et al. (44) have shown that heat stress can significantly decrease milk production in dairy cows. Another research highlighted the sensitivity of dairy cows to high temperatures and humidity, which affects feed intake, reproductive performance, and milk production (8). Therefore, although an increase in milk production with the presence of ventilation systems was observed under alert conditions, it may be relatively less effective in these climatic conditions.

Feed costs also played an important role in the economic outcomes. The greatest increase in intake occurred at THI-1 (0.46 kg/cow/day), which led to both higher feed expenses and higher milk production. At THI-2 and THI-3, the more modest increases in feed intake (0.24 kg/day and 0.30 kg/day, respectively) indicate that cows were less efficient at metabolizing the additional energy supplied by the increased milk production under more extreme heat stress. This is consistent with longstanding evidence correlating heat stress with decreased feed efficiency in dairy cows (45, 46). According to recent findings, DMI and THI have a generally unfavorable connection. West et al. (47) reported a substantial reduction of approximately 0,51 kg DMI for every unit increase in mean THI values on the same day. A plateau for DMI was assessed by Gorniak et al. (48) further observed that DMI remains at a plateau until THI reaches 60. Feed consumption decreased by approximately 1 kilogram per day after that. However, it has also been mentioned that the decrease in milk production in heat-stressed dairy cows is not entirely attributable to the decrease in feed consumption (49). Energy consumption constituted the largest expense, with energy costs declining from 108.67€/year/fan at THI-1 to 60.65€/year/fan at THI-3. This reduction in energy costs may be explained by fewer hours of fan operation or by reduced cooling demand at higher THI levels, where local environmental conditions may have limited the cooling capacity of the fans.

The performance of ventilation systems in different climatic zones can be evaluated using sensitivity analysis, as shown in previous research (50). Sensitivity analysis allows investment decisions to be made more consciously and strategically and helps farmers evaluate how ventilation systems will perform under different climatic and economic conditions (51). In our study, sensitivity analysis revealed that increases in milk prices led to a higher B/C under all conditions. At the current milk price of 0.401 €/kg, ventilation systems yielded the highest B/C ratio under discomfort conditions and the lowest B/C ratio under alert conditions. It is important for farmers to observe economic conditions periodically and develop strategic plans against cost management and price fluctuations. While the current scenarios focus on favorable changes, the analysis could equally be extended to include adverse conditions, such as decreases in the milk price or increases in input costs, as well as additional variables such as labor costs, equipment depreciation, or changes in ventilation duration, to explore a broader range of potential outcomes.

The breakeven price of milk is the price at which incremental milk production is profitable and offsets the costs of the cooling system (24). In our study, the milk price was 0.401 €/kg and was compared to the breakeven price at different THI levels. With a breakeven price of 0.30€/kg at THI-1, the current milk price is sufficient to cover costs and generate a profit, highlighting favorable conditions for milk production. Similarly, at THI-2, the breakeven price further declined to 0.26 €/kg, reinforcing the economic advantage of the ventilation system even under discomfort conditions. However, at THI-3, the breakeven price increased to 0.32 €/kg, narrowing the profit margin as thermal stress intensifies. Nonetheless, since the current milk price (0.401 €/kg) still remains above this threshold, the system continues to ensure profitability even during periods of severe heat stress.

In addition, the income over feed costs (IOFC) highlights the economic consequence of cooling systems across various THI ranges. The IOFC was highest at THI-1 (6.56€/day/cow), followed by THI-2 (6.47€/day/cow) and THI-3 (6.34€/day/cow), which indicated a gradual decrease as environmental stress increased. These values refer to cows under ventilation (with VSs), while IOFC values were lower in all THI groups without ventilation systems, confirming the economic advantage of cooling under moderate heat stress. This trend implies that, although the effect of cooling systems promotes feed intake and conversion under low/medium heat stress, the effective contributions of cooling systems become limited under intense thermal stress. On the other hand, our partial budget analysis excluded reproductive and health benefits that ventilation may provide via heat-stress mitigation. Therefore, the operational IOFC likely underestimates the total benefits. These results highlight the need for the coupling of cooling systems with customized nutritional interventions, such as high-energy feeding, to maximize feed utilization and profitability during stressful periods.

Our results indicate that the efficacy of ceiling fans can be consistently relied upon during mild tomoderate heat stress, whereas severe heat stress probably requires a combination of fans + water strategies (52). A next step may be to integrate this economic analysis with precision livestock farming (PLF) streams to measure heat exposure where it matters—at the pen/cow level—monitor fast physiological and behavioral responses to such heat exposure, and update the IOFC in near real time (53). For example, wearable sensors (activity, lying time, rumination collars, or ear-tags) and high-resolution indoor climate arrays (T/RH/air speed by zone) could mitigate misclassification bias associated with outdoor daily THI and explain milk yield responses under moderate heat stress (54). Computer-vision pipelines can quantify bunk attendance, time spent under fans, crowding, and respiration/panting proxies, and these signals can trigger cost-aware control (55). This PLF integration would improve causal interpretation by observing proximal mediators rather than relying solely on calendar adjustments (56).

In short-term applications, high fixed costs and limited returns may reduce the net economic impact and B/C (57). In long-term applications, increasing total returns and spreading costs over a longer period of time may enhance the net economic impact and B/C (58). Therefore, dairy farms can maximize the return on investment (ROI) by using ventilation systems for longer periods. Further studies can focus on the long-term use of cooling systems to monitor the return on investment.

There are some drawbacks to partial budget analysis. Some cost or income factors can be overlooked, which may pose a problem. Therefore, it is necessary to expand the scope of the analysis and include missing cost factors. These factors can affect the overall cost structure of the farm and may play an important role in farmers’ decision-making process (59). The partial budget’s inability to display the full breakeven point for dairy farms is also another important issue. In some situations, the farmer wants to know how a particular decision will affect the bottom line. For example, the breakeven milk price is only marginally helpful to farmers; in these situations, the total breakeven point would probably have greater importance. Therefore, it is recommended that further studies conduct a more comprehensive economic analysis under different scenarios by expanding the scope of the data.

## 5 Conclusion

Farms should plan their investments in ventilation systems by taking into account climate conditions and the relative humidity index. In our study, it was determined that investments made under discomfort conditions (THI-2) provided higher economic returns (B/C ratio); therefore, investments in regions with such conditions may be more profitable. In addition, the use of ventilation systems increased milk production and improved key reproductive parameters such as days open and number of services per pregnancy, but it failed to fully counteract the detrimental impacts of severe heat stress. Similarly, ventilation systems increased milk yield; however, their costs and inability to cause a considerable change in milk composition under severe conditions make economic returns context-dependent. Sensitivity analyses also showed that profitability could be enhanced by increasing the milk price or reducing operational costs, such as energy and feed. These results once again confirm the relevance of strategic implementation and the potential to combine environmental management technologies to maximize effectiveness and economic viability. In conclusion, ventilation systems are among the useful tools that improve the productivity of dairy cows under heat stress; however, their decreased efficiency under extreme conditions highlights the need for a multidimensional approach. This study focused on short-term operational economics (milk revenue vs. operating costs), while capital expenditures and long-term maintenance were beyond its scope. Future directions should focus on the integration of these systems with advanced cooling technologies, expanded cost and benefit factors, energy-efficient designs, and nutritional strategies to enhance their impact. This multidimensional approach promotes sustainability in herd management by reducing heat stress losses, improving energy efficiency and resource use, and, at the same time, safeguarding animal welfare and farm economic viability under increasing climate variability.

## Data Availability

The raw data supporting the conclusions of this article will be made available by the authors, without undue reservation.
