# Supplementary material for: Integrative assessment of the effects of ventilation systems on economic efficiency, milk production, and reproductive performance in dairy cows
Source: Front Vet Sci. 2025 Dec 4;12:1713828. doi: 10.3389/fvets.2025.1713828 (PMC12711547; doi:10.3389/fvets.2025.1713828)

**Supplementary Figure 1.** Schematic representation of the monitoring periods in all 4 sampled dairy farms before and after installation of ceiling fans (represented by crosses).


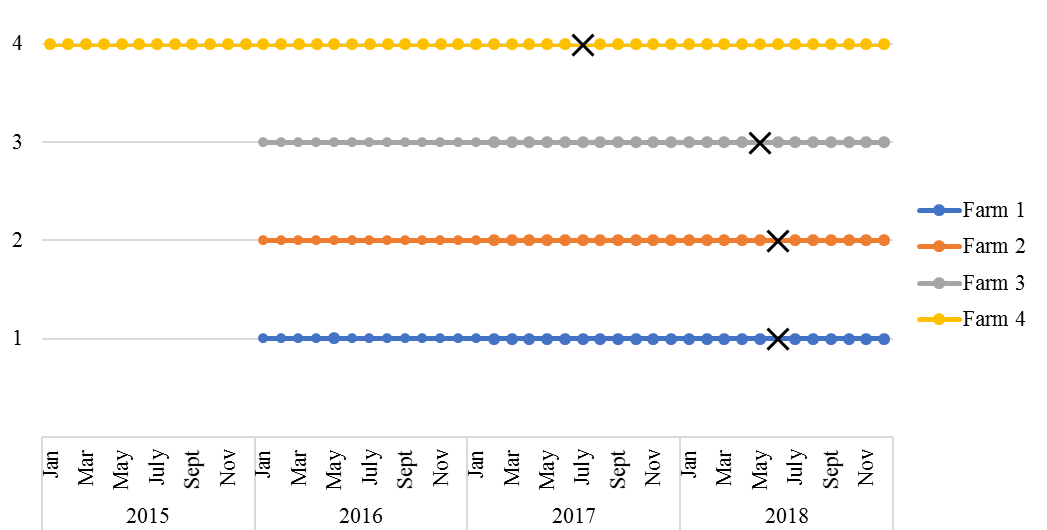


**Supplementary Figure 2.** Trends in average maximum temperature and relative humidity across the years 2015–2018.


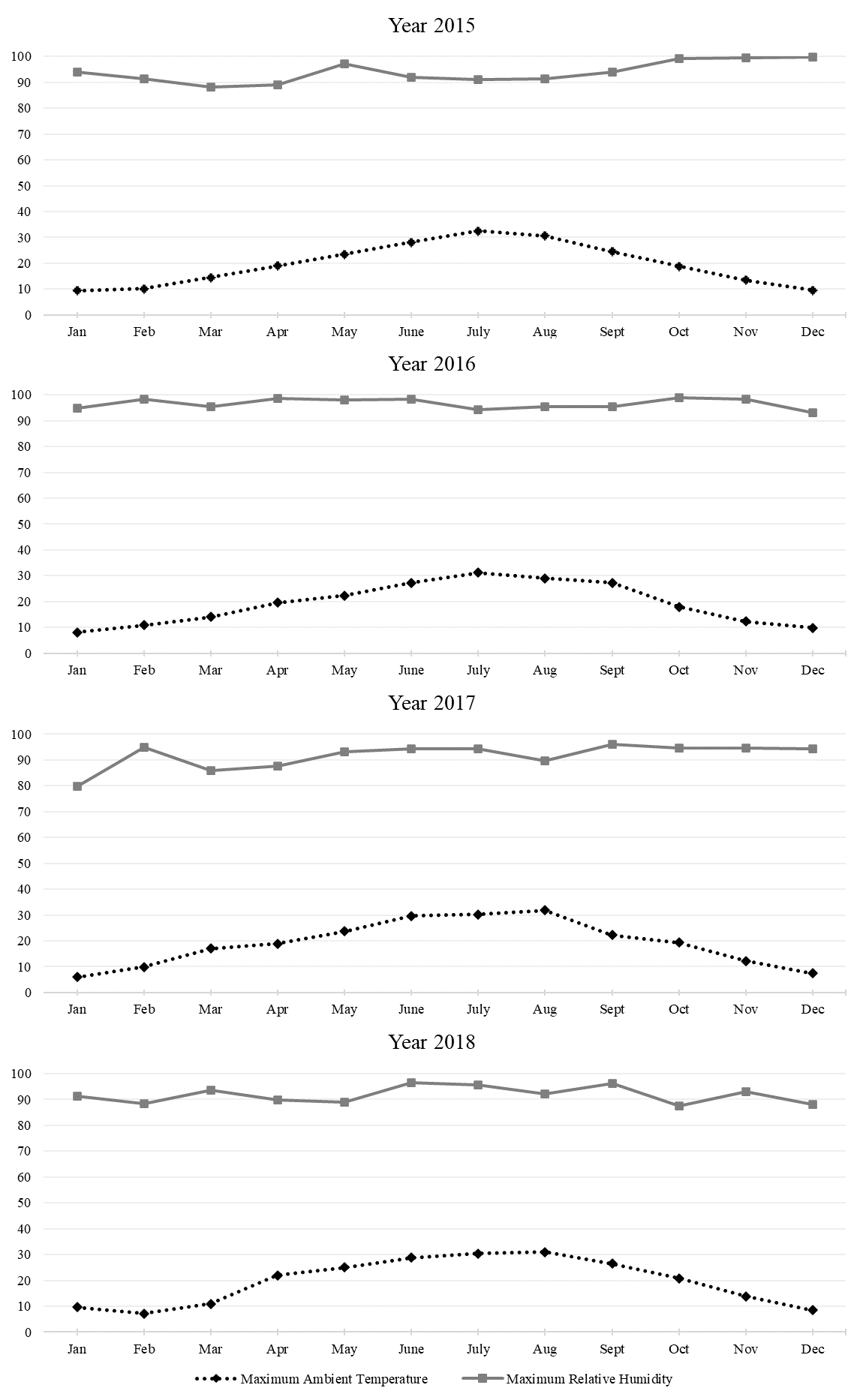

Supplement: Supplementary file 1 [file Data_Sheet_1.docx]
